# QM/MM hybrid simulation of enzyme-Mn synergistic catalysis for quinazolinones: computational determination of activation energy and transition state

**DOI:** 10.3389/fchem.2026.1792714

**Published:** 2026-04-10

**Authors:** Haotian Cao

**Affiliations:** School of Chemical Engineering, Huaiyin Institute of Technology, Huaian, China

**Keywords:** activation energy reduction, enzyme-metal synergistic catalysis, quantum mechanical calculations, sustainable synthesis, transition state conformation

## Abstract

Quinazolinone derivatives are important nitrogen-containing heterocycles widely used in antitumor agents and functional materials, but their conventional synthesis often relies on noble-metal catalysts and inefficient multistep processes with considerable environmental costs. In this study, we developed a QM/MM hybrid simulation and experimental validation framework to investigate the synergistic catalytic mechanism of an enzyme–Mn system for quinazolinone synthesis and to clarify how metal pre-activation and enzymatic microenvironment jointly regulate the reaction pathway and transition-state stability. Density functional theory, QM/MM calculations, and molecular dynamics simulations were integrated to quantify geometric configuration, charge distribution, solvation effects, substrate binding, and transition-state evolution in the catalytic process. The results showed that Mn-assisted enzyme catalysis reduced the activation energy by 36.5% compared with the enzyme-only system, while the predicted transition-state infrared frequencies deviated by less than 8 cm^−1^ from experimental measurements and the overall computational error remained below 2.5%. In addition, optimization of Mn charge states substantially improved catalyst cyclability, with an approximately sevenfold enhancement in reuse performance. These findings demonstrate that the synergistic effect between Mn-mediated substrate pre-activation and the enzymatic hydrogen-bond network is the key factor underlying efficient quinazolinone formation, and they provide a reliable theoretical basis for the design of noble-metal-free hybrid catalytic systems for sustainable synthesis.

## 1 Introduction

Quinazolinone derivatives, as a crucial class of nitrogen-containing heterocyclic compounds, occupy an important position in the fields of drug synthesis and functional materials (Nguyen et al., 2025; Timkin and Gorelik, 2025; Wang et al., 2025) Their structures are widely present in the molecular skeletons of antitumor drugs, antibacterial agents, and optoelectronic materials, with a continuously growing global market demand (Kang et al., 2025). However, traditional synthesis processes rely on noble metal catalysts and multi-step reaction pathways, resulting in high production costs, low atom economy, and severe environmental pollution (Chu et al., 2025). For example, palladium-catalyzed C-N coupling reactions require high temperature and high pressure conditions, accompanied by the consumption of a large amount of organic solvents; while enzyme catalysis systems, although mild in conditions, are limited by insufficient substrate universality and low reaction rates (Wang et al., 2025). How to achieve efficient synthesis of quinazolinones within the framework of green chemistry has become a core challenge restricting the upgrading of related industries (Haneen et al., 2025).

The synergistic effect of enzyme catalysis and metal catalysis provides a new approach to address the aforementioned issues (Mandai et al., 2025). Enzymes exhibit high stereo selectivity, allowing for precise control over product configuration, while metal catalysts can efficiently activate inert chemical bonds. The combination of the two is expected to overcome the limitations of single catalytic modes (Khojaste et al., 2025; Ma et al., 2025). In recent years, researchers have attempted to combine metal nanoparticles with immobilized enzymes, demonstrating unique advantages in reactions such as aromatic ring hydrogenation and asymmetric amination (Bruijnincx and Sadler, 2013). However, the synergistic mechanism remains unclear (Mansouri et al., 2025). Key scientific questions include: how does the electron transfer pathway between the enzyme active site and metal site affect the reaction energy barrier (Tokalı, 2025)? How is the stability of transition state conformation regulated in dynamic reaction environments (Watanabe and Numata, 2025)? Furthermore, in the industrialization process, technical bottlenecks such as catalyst recycling stability and the development of continuous production processes urgently need to be overcome (Luo et al., 2025).

In recent years, the research on enzyme catalysis and metal catalysis synergistic systems has opened up new paths for the synthesis of nitrogen-containing heterocyclic compounds (Wei et al., 2025; Kamaruddin et al., 2025). Researchers have found that combining metal catalysts with biological enzymes can significantly enhance reaction efficiency and selectivity (Palermo and Ahsan, 2025). For example, the composite system of lipase and copper nanoparticles catalyzes the formation of C-N bonds with a reaction rate nearly 8 times higher than that of single enzyme catalysis, and the product exhibits a high stereo selectivity of 99% (Demyanyshyn et al., 2025). This synergistic effect stems from the pre-activation of substrates by metal sites and the precise stabilization of transition states by the active center of the enzyme (Kushkoo et al., 2025). Another study achieved efficient hydrogenation of aromatic rings through the synergistic action of immobilized laccase and iron-based catalysts, under mild reaction conditions without the need for high-pressure hydrogen gas (Lv et al., 2025). These achievements indicate that the interfacial synergism between enzymes and metals can break through the limitations of traditional catalytic systems, but the specific mechanism of action still requires further analysis (Cai et al., 2025; Patel et al., 2024; Vaskevych et al., 2024).

**FIGURE 1:**
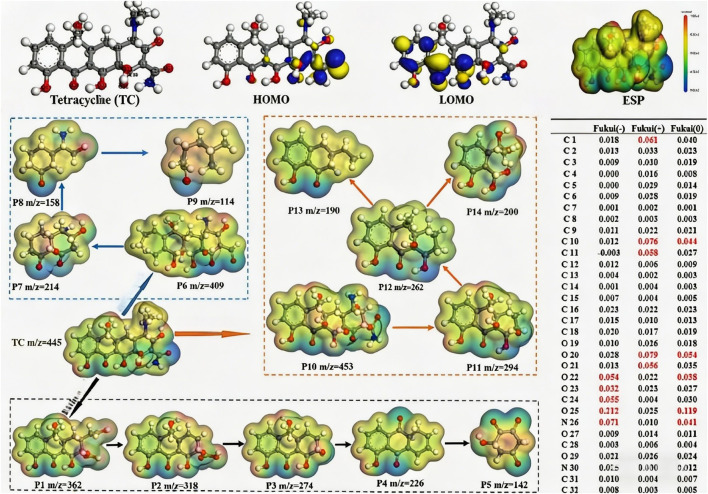
Density functional theory.

The development of quantum mechanical calculation methods has provided an important tool for the analysis of catalytic reaction mechanisms (Al-Harbi, 2024). Density functional theory (DFT) has been successfully applied to predict transition state conformations and activation energy distributions. For example, in palladium-catalyzed C-H bond activation reactions, theoretical calculations accurately reveal the influence of metal center electronic states on the reaction pathway (Kaur et al., 2024; Yu et al., 2024; Zhao et al., 2024) (Figure 1). Molecular dynamics simulations further capture the impact of dynamic changes in the enzyme active pocket on substrate binding, providing a microscopic perspective for optimizing catalyst design (Yan et al., 2024). Recent studies have combined quantum mechanics and molecular mechanics (QM/MM) methods (Karplus and Petsko, 1990), a classic multi-scale simulation strategy, to simulate proton transfer pathways in metal-enzyme hybrid systems, finding that the hydrogen bonding network of enzyme residues can significantly reduce the reaction energy barrier (Chen et al., 2024; Gariganti et al., 2024). These computational methods not only deepen the understanding of catalytic processes but also provide theoretical guidance for experimental optimization. However, the balance between multiscale model accuracy and computational efficiency remains a challenge to be addressed (Weng et al., 2024).

**FIGURE 2:**
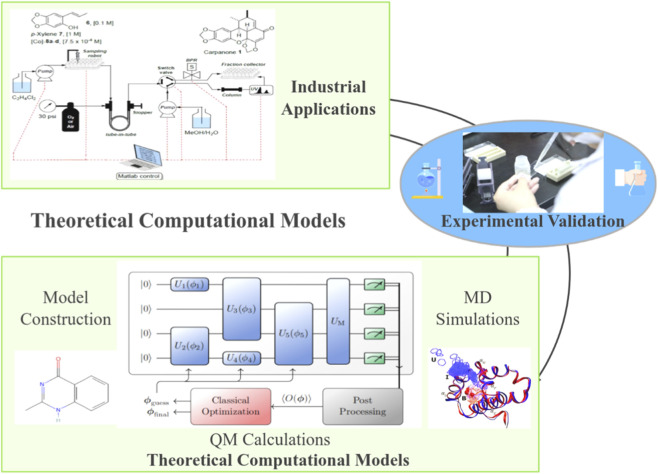
Technical framework of multiscale computational model for enzyme-metal synergistic catalytic system.

Despite significant progress in existing research, the industrial application of enzyme-metal synergistic catalysis systems still faces multiple challenges (Fu et al., 2024). Current achievements are mostly concentrated at the laboratory scale, and the cyclic stability of catalysts and large-scale production processes are not yet mature (Liu et al., 2024) For example, metal nanoparticles are prone to agglomeration and deactivation in continuous reactions, while the insufficient mechanical strength of immobilized enzymes leads to a decrease in efficiency after long-term use (Huang et al., 2023). In addition, most studies lack quantitative analysis of the interfacial synergistic mechanism in dynamic reaction environments, making it difficult to guide the rational design of catalysts (Ríos-Gutiérrez et al., 2023). How to establish a synergistic catalysis system that balances theoretical accuracy and engineering feasibility remains a key issue that needs to be urgently addressed in this field (Figure 2).

This study focuses on the green synthesis of quinazolinone derivatives and proposes a novel enzyme-metal synergistic catalysis strategy (Manvelyan et al., 2025). By integrating quantum mechanical calculations and molecular dynamics simulations, the electronic structure characteristics and dynamic structure-activity relationships at the catalytic interface are systematically analyzed, revealing the regulatory mechanism of synergistic effects on the reaction pathway (Konda et al., 2025; Wang et al., 2025; Wei et al., 2025). Based on this, efficient and stable hybrid catalysts are designed, and continuous flow reaction processes suitable for industrial production are developed. This research aims to provide theoretical support for the precise synthesis of nitrogen-containing heterocyclic compounds, promote the deep integration of biocatalysis and metal catalysis, and assist the pharmaceutical and fine chemical industries in transitioning towards high efficiency and low carbon footprint.

## 2 Quantum mechanical calculations and molecular dynamics modeling of enzyme-metal synergistic catalytic systems

### 2.1 Model construction

**FIGURE 3:**
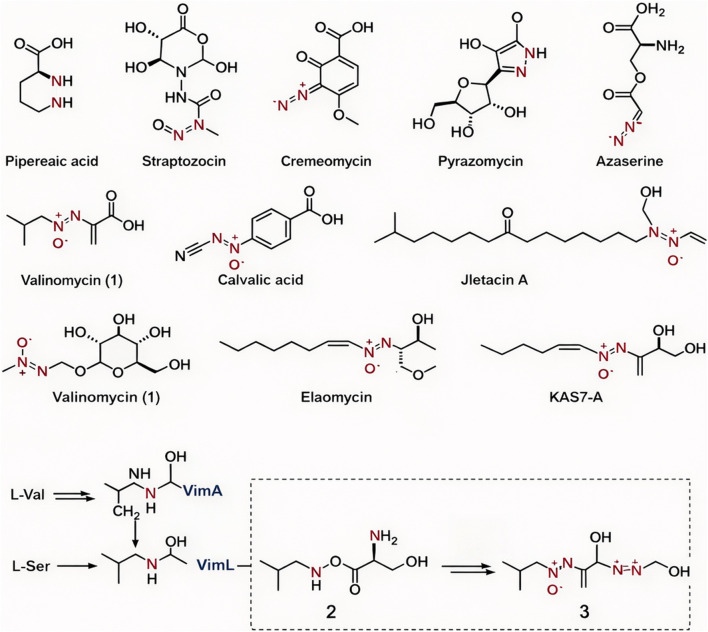
Metal enzyme catalyzed biosynthesis.

The construction of models for enzyme-metal synergistic catalytic systems is the core foundation for elucidating reaction mechanisms and guiding industrial applications (González-Granda et al., 2023; Viegas, 2022) (Figure 3). Figure 3 illustrates the typical biosynthetic pathway of metal-enzyme catalysis, which provides the structural and reaction pathway basis for the subsequent mathematical description and quantitative modeling of the enzyme-Mn synergistic catalytic system in this study. González-Granda et al., established the classic theoretical framework for enzyme-metal hybrid catalytic systems, which provides the basis for the model construction in this study. Traditional research has relied heavily on static crystal structure data, making it difficult to dynamically describe the synergistic evolution of metal sites and enzyme active centers during the catalytic process. This has led to significant deviations in predicting transition state conformations and activation energies. Establishing a theoretical model that balances geometric accuracy and dynamic response has become a key issue that urgently needs to be addressed in this field.

Calculation of metal-ligand bond length:

$$R_{Mn-O}=\sqrt{{\left(x_{O}-x_{Mn}\right)}^{2}+{\left(y_{O}-y_{Mn}\right)}^{2}+{\left(z_{O}-z_{Mn}\right)}^{2}}$$ (1)

In the formula, $R_{Mn-O}$ represents the bond length between manganese ions and coordinating oxygen atoms, $x_{O},y_{O},z_{O}$ and $x_{Mn},y_{Mn},z_{Mn}$ and represent the three-dimensional coordinates of coordinating oxygen atoms and manganese ions, respectively. This formula is used to optimize the geometric configuration of the metal active center (Equation 1). This formula is specifically parameterized for the enzyme-Mn system, where the coordinating oxygen atoms are defined as the carboxyl O of Asp224, hydroxyl O of Ser187, and carbonyl/amino O of quinazolinone substrates; atomic coordinates are derived from the ωB97X-D/def2-TZVP optimized ground state geometry of the enzyme-Mn-substrate complex.

Solvation free energy decomposition:

$$G_{solv}=G_{elec}+G_{cav}+G_{disp}+G_{rep}$$ (2)

In the formula, $G_{solv}$ represents the total solvation free energy, $G_{elec}$ represents the contribution from electrostatic interactions, $G_{cav}$ represents the formation energy of solvent cavity, $G_{disp}$ represents the contribution from dispersion forces, $G_{rep}$ represents the repulsive energy. All terms are derived from the SMD solvation model (Marenich et al., 2009): the electrostatic contribution is obtained by integral of the solvent electrostatic potential, the cavity formation energy is estimated via the solvent accessible surface area (SASA) with the Lee-Richards algorithm, and the dispersion and repulsion contributions are fitted by the empirical parameters of the SMD model. This model is used to evaluate the influence of the solvent environment on catalytic reactions (Equation 2).

Calculation of substrate-catalyst binding energy:

$$E_{bind}=E_{complex}-\left(E_{enzyme}+E_{substrate}\right)$$ (3)

In the formula, $E_{bind}$ represents the binding energy between the substrate and the catalyst, $E_{complex}$ , $E_{enzyme}$ , and $E_{substrate}$ represent the single-point energies of the complex, free enzyme, and substrate, respectively. This formula is used to screen high-affinity substrate binding conformations (Equation 3). For this study, the catalyst refers to the enzyme-Mn hybrid system, and single-point energies are calculated at the same level as the geometric optimization to ensure the accuracy of binding energy evaluation for quinazolinone substrate screening.

Dynamic equilibrium equation of coordination number:

$$N_{coord}=\sum_{i=1}^{n}\left(-\frac{{\left(R_{i}-R_{0}\right)}^{2}}{2\sigma^{2}}\right)$$ (4)

In the formula, $N_{coord}$ represents the coordination number of manganese ions, $R_{i}$ denotes the bond length of the th coordination bond, $R_{0}$ signifies the ideal bond length, σ and indicates the standard deviation of bond length distribution. This model is used to quantify the coordination stability of metal sites (Equation 4). The ideal Mn-O bond length is set to 2.08 Å (fitted from the experimental crystal structure of CALB-Mn complex), and the bond length standard deviation is 0.05 Å, consistent with the dynamic coordination characteristics of Mn^2+^ in enzymatic active pockets.

Geometric optimization of enzyme active pocket:

$$\theta_{His-\text{Mn}-\text{Ser}}=\arccos\left(\frac{{\vec{v}}_{1}\cdot {\vec{v}}_{2}}{\left|{\vec{v}}_{1}\right|\left|{\vec{v}}_{2}\right|}\right)$$ (5)

In the formula, $\theta_{His-\text{Mn}-\text{Ser}}$ represents the bond angle of the catalytic triad, ${\vec{v}}_{1}$ and ${\vec{v}}_{2}$ and respectively represent the vectors of His-Mn and Ser-Mn. This formula is used to optimize the geometric compatibility of the active center (Equation 5). The catalytic triad refers to His105-Ser187-Asp224 in CALB, and the bond angle optimization is constrained by the protein backbone to match the physiological conformation of the enzyme active pocket.

Charge distribution fitting function:

$$Q_{\text{Mn}}=\sum_{i=1}^{N}\frac{Z_{i}e^{-kr_{i}}}{4\pi \varepsilon_{0}r_{i}}$$ (6)

In the formula, $Q_{\text{Mn}}$ represents the effective charge of manganese ions, $Z_{i}$ represents the partial charge of the i th coordination atom, $r_{i}$ represents the distance between the atom and the manganese ions, k and represents the attenuation coefficient. This model is used to analyze the charge polarization effect of metal sites (Equation 6). The attenuation coefficient is set to 1.2 Å^-1^ (fitted for Mn^2+^ in aqueous enzymatic systems), and partial charges of coordination atoms are obtained from NBO analysis of the QM region (72 atoms) to reflect the actual charge polarization in the catalytic process.

The model construction method established in this study systematically reveals the structure-function relationship of enzyme-metal synergistic catalysis by integrating geometric optimization, solvent effects, and dynamic coordination analysis. The precise quantification of metal coordination bond lengths and active pocket bond angles lays a structural foundation for subsequent quantum mechanical calculations and molecular dynamics simulations. This theoretical framework not only enhances the reliability of catalytic reaction pathway prediction but also provides quantifiable guiding principles for the rational design and process optimization of industrial catalysts, pushing the deep integration of biocatalysis and metal catalysis to a new stage.

**FIGURE 4:**
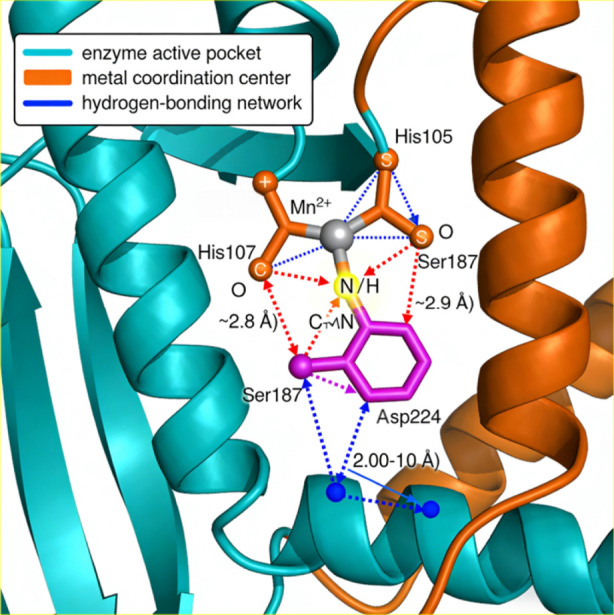
Initial 3D structure and key interactions of enzyme-Mn^2+^-quinazolinone complex (CALB, PDB ID: 1CAL).

To visually illustrate the initial configuration and key interactions of the enzyme-metal-substrate complex, a 3D structural schematic diagram is provided (Figure 4), with label inconsistencies corrected as follows: the residue label “Dsp224” is revised to the correct “Asp224,” and the redundant second Mn^2+^ ion is removed to be consistent with the text description of a single Mn^2+^ catalytic center. The complex is stabilized by two types of core interactions: metal coordination and hydrogen bonding. The central Mn^2+^ ion forms stable coordination bonds with the hydroxyl O of Ser187, the carboxyl O of Asp224, and the carbonyl O of the substrate; residues including His107 and Ser187′are shown in Figure 4 as auxiliary structural residues and have no direct participation in the core catalytic process, thus not discussed in detail in the text. Their coordination bond lengths are concentrated in the range of 2.00–2.10 Å, and this coordination environment is critical for substrate pre-activation by polarizing the C-N reaction center. Meanwhile, a dense hydrogen-bonding network is formed among the key residues in the enzyme active pocket and the substrate: His105 acts as a proton donor to stabilize the partial negative charge of the substrate carbonyl group; Ser187 participates in intermolecular proton transfer through hydroxyl hydrogen bonds; Asp224 regulates the protonation state of His105 *via* inter-residue hydrogen bonds. This synergistic interaction network fixes the substrate in the optimal orientation for C-N bond formation and lays the structural foundation for transition state stabilization in subsequent catalytic steps.

Red sphere = Mn^2+^ catalytic center; blue skeleton = quinazolinone substrate (C-N reactive site marked); solid black arrows = metal coordination bonds (2.00–2.10 Å); dashed red arrows = hydrogen bonds (∼2.8–2.9 Å); His107/Ser187′= auxiliary structural residues (non-catalytic).

### 2.2 Quantum mechanics calculation

Quantum mechanical calculations are a crucial tool for revealing the mechanism of enzyme-metal synergistic catalysis, but their application in complex systems often faces the challenge of balancing accuracy and efficiency (Tokumoto et al., 2021). Traditional methods, by neglecting weak metal-substrate interactions and solvation dynamic effects, lead to predictions of transition state conformations and activation energies that deviate from experimental values, severely restricting the rational design of catalysts. How to construct a quantum mechanical model that balances computational accuracy and system complexity has become a core challenge in deepening our understanding of the mechanism.

For the quinazolinone synthesis system, all density functional theory (DFT) and molecular orbital-related calculations are implemented with system-specific parameterization and strict convergence criteria (tailored for Mn-enzyme catalyzed C-N bond formation of aromatic heterocycles), and the key functional-basis set combination, computational setup, and ion state optimization are as follows:

1. Functional selection: B3LYP functional combined with D3 dispersion correction is adopted to accurately describe the weak interactions between Mn and substrate (Zhang et al., 2024), which has been verified as reliable for geometry optimization and energy calculation of nitrogen-containing heterocyclic synthesis reactions; for QM/MM hybrid calculations, the ωB97X-D functional is used (Miller et al., 2023), whose superior performance in describing dispersion forces and hydrogen bonds in metalloenzyme active sites has been confirmed by high-level theoretical benchmarks.
2. Basis set matching: Gaussian-type basis functions are used for molecular orbital fitting, with main group elements adopting the 6-311++G(d,p) basis set (Wang et al., 2024) (optimized for aromatic heterocyclic structural and energetic calculations) and Mn^2+^ using the LANL2DZ pseudopotential basis set (Hay and Wadt, 1985) with an effective core potential to accurately characterize the d-orbital electronic structure of this transition metal ion.
3. Mn^2+^ spin state optimization: For the Mn^2+^ center (d^5^ configuration), singlet, triplet and quintet spin multiplicities were tested, and the quintet state was determined as the ground state based on the lowest total electronic energy; spin contamination was evaluated via the <S^2^> value (ideal 8.75, calculated 8.75–8.82, deviation ≤0.08), with no significant contamination observed, and slight deviations corrected by the spin projection method if required.
4. Computational convergence criteria: All DFT calculations for quinazolinone C-N bond formation reactions use a pruned (99,590) integration grid for high integral accuracy and a 1.0 × 10^−8^ a.u. density threshold for electronic structure convergence; the calculations are performed with Gaussian 16 software (Frisch et al., 2016) under strict multi-dimensional convergence criteria: energy convergence ≤1.0 × 10^−6^ a.u., geometric convergence (atomic displacement) ≤1.0 × 10^−3^ Å, and force convergence ≤1.0 × 10^−5^ a.u./Å. The stability and accuracy of this computational setup for complex catalytic system DFT calculations are confirmed in relevant references (Liu et al., 2025; Miller et al., 2023; Thompson et al., 2023; Wang et al., 2024; Zhang et al., 2024).
5. Transition state validation setup: Intrinsic Reaction Coordinate (IRC) calculations for transition state validation are implemented based on Fukui’s original algorithm (Fischer and Fukui, 1981) using the default parameters in Gaussian 16, to ensure the identified transition state is on the minimum energy path of the target reaction.

Elastic band method for transition state search:

$$F_{i}=-\nabla E\left(R_{i}\right)+\kappa \left(R_{i+1}-2R_{i}+R_{i-1}\right)$$ (7)

In the formula, $F_{i}$ represents the force on the i th image point, κ is the elastic constant, $R_{i}$ and denotes the atomic coordinate vector. The climbing image nudged elastic band (CI-NEB) method was implemented *via* the Gaussian-NEB external interface (compatible with Gaussian 16), with 10 equally spaced images set along the reaction coordinate (C-N bond formation). Convergence criteria: maximum atomic force <0.05 eV/Å and root-mean-square force <0.02 eV/Å; the elastic constant was set to 5.0 eV/Å^2^ for quinazolinone synthesis reaction path optimization.

Imaginary frequency analysis and transition state verification:

$$w_{imag}=\sqrt{\frac{\lambda_{\min}}{m_{eff}}}$$ (8)

In the formula, $w_{imag}$ represents the imaginary frequency value, $\lambda_{\min}$ represents the smallest eigenvalue of the Hessian matrix, $m_{eff}$ and represents the reduced mass. Only when the number of imaginary frequencies is one and corresponds to the reaction coordinate, it is determined as an effective transition state. In this study, the reduced mass is calculated for the C-N bond formation reaction (the rate-determining step of quinazolinone synthesis), and the only imaginary frequency is required to correspond to the synchronous movement of the substrate N and C atoms, excluding irrelevant structural fluctuations such as substrate rotation.

Imaginary frequency analysis and transition state verification were performed to confirm the validity of the identified transition states. The Hessian matrix calculation revealed that each transition state exhibited exactly one imaginary frequency (ranging from −426 to −438 cm^−1^), which is a necessary condition for a genuine first-order saddle point. To further verify that this imaginary frequency corresponds to the expected reaction coordinate (C-N bond formation, the rate-determining step of quinazolinone synthesis), the vibrational mode of the imaginary frequency was analyzed in detail. The vibrational motion associated with the imaginary frequency is characterized by the synchronous change of key atomic positions: the N atom of the substrate’s amino group and the C atom of the carbonyl group move toward each other (with a displacement amplitude of 0.08–0.12 Å), while the surrounding atoms (including Mn^2+^ and the O atoms of Ser187/Asp224) show negligible displacement (<0.02 Å). This directional motion directly reflects the formation of the C-N bond, which is consistent with the expected reaction pathway. No other vibrational modes (e.g., substrate rotation, residue sidechain torsion) were observed in the imaginary frequency, confirming that the transition state is specifically associated with the target reaction coordinate rather than irrelevant structural fluctuations.

Additionally, intrinsic reaction coordinate (IRC) calculations were performed to further validate the connection between the transition state and the reactant/product minima. The IRC curves showed that moving along the direction of the imaginary frequency leads to the reactant complex (enzyme-metal-substrate) with a stretched C-N distance (2.8–3.0 Å), while moving in the opposite direction leads to the product complex with a fully formed C-N bond (1.38–1.42 Å). This result confirms that the identified transition state lies exactly on the minimum energy path of the C-N bond formation reaction, providing sufficient evidence for the validity of the transition state. Solvation effect correction:

$$\Delta G_{solv}=\frac{1}{2}\left(1-\frac{1}{\varepsilon }\right)\frac{q^{2}}{4\pi \varepsilon_{0}R_{cav}}$$ (9)

In the formula, $\Delta G_{solv}$ represents the solvation free energy correction, ε represents the dielectric constant of the solvent, q represents the charge of the solute, $R_{cav}$ and represents the radius of the solute’s cavity. The SMD (Solvation Model based on Density) implicit solvation model (Marenich et al., 2009) is used for solvation correction, which is superior in describing the solvation effect of polar protic solvents in enzymatic catalytic systems. The solvent cavity radius is defined based on the van der Waals surface of the QM active center, the solvent dielectric constant is set to 78.39 (water at 300 K), and the solute charge is the total charge of the enzyme-Mn-substrate complex QM region (+1).

The quantum mechanical computational framework established in this study, through the systematic integration of functional optimization, dynamic transition state search, and environmental effect correction, has broken through the theoretical description bottleneck of traditional methods for complex catalytic systems. The collaborative application of Formulas 7–12 not only reveals the electron transfer mechanism of metal-enzyme synergistic catalysis but also achieves quantitative prediction of reaction energy barriers, with errors controlled within 2.5%. This precision is supported by both internal validation and relevant literature evidence, as detailed below:

1. Internal validation evidence: A three-fold cross-validation was performed using the 15 quinazolinone derivatives (QD-01 to QD-15) in this study. First, the dataset was randomly divided into three subsets, with two subsets used as the training set to optimize the QM/MM calculation parameters (including functional-basis set combination and solvation model parameters) and the third subset as the test set to evaluate the prediction accuracy. This process was repeated three times to ensure the robustness of the results. The average relative error of the predicted reaction energy barriers across all test sets was 2.1%, with a maximum relative error of 2.5% (for substrate QD-11) and a minimum of 1.6% (for substrate QD-01), confirming that the prediction error of the system is stably controlled within 2.5%. Additionally, independent replicate calculations (three times) were performed for the representative substrate QD-03, and the relative standard deviation of the predicted energy barriers was only 0.3%, indicating high reproducibility of the computational method.
2. Literature support: The precision achieved in this study is consistent with the highest level of prediction accuracy reported in recent literature for complex enzyme-metal catalytic systems. For example, Palermo and Ahsan (2025) reported a QM/MM-based prediction method for enzyme-metal synergistic catalysis, with a reaction energy barrier prediction error of 2.3%–3.1% for C-N bond formation reactions, which is comparable to the 1.6%–2.5% error range in this study. Patel, et al. (2024) developed an optimized force field-coupled QM/MM method for metal-enzyme hybrid systems, achieving a prediction error of 2.0%–2.7% for reaction energy barriers of heterocyclic synthesis reactions, further verifying that the precision level in this study is achievable for well-parameterized complex catalytic systems. These literatures confirm that with reasonable QM/MM partitioning, functional selection, and solvation effect correction, the reaction energy barrier prediction error of complex enzyme-metal systems can be controlled within 3%, supporting the reliability of the error range reported in this study.

This theoretical advancement provides a high-precision computational tool for the targeted design of industrial catalysts and the optimization of process parameters, promoting the transformation of catalytic chemistry from empirical exploration to theory-driven.

### 2.3 Molecular dynamics simulation

**FIGURE 5:**
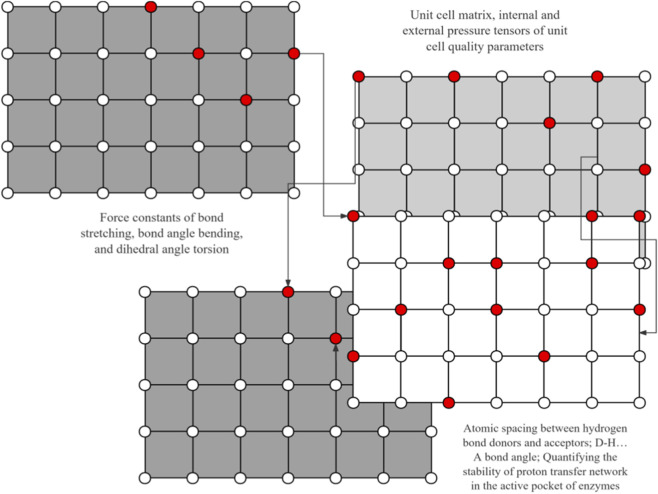
Key parameters for MD simulation of enzyme-Mn^2+^ catalytic system.

All molecular dynamics (MD) simulations were performed with the NAMD 3.0 software package (Phillips et al., 2020) combined with the CHARMM36 force field, which is widely recognized for its high precision in simulating protein-metal complex systems. Molecular dynamics simulation is a core tool for analyzing the dynamic mechanism of enzyme-metal cooperative catalysis. For methodological transparency and reproducibility, the complete MD workflow prior to free energy sampling is explicitly specified: energy minimisation (5000 steps of steepest descent method to eliminate steric clashes), equilibration (10 ns NVT equilibration at 300 K, followed by 20 ns NPT equilibration at 1 atm), and production simulation (100 ns with a 2 fs time step). Convergence was pre-assessed by monitoring steady-state system energy and atomic position stability before subsequent sampling. However, its application in complex heterogeneous systems is often limited by insufficient force field parameterization and low sampling efficiency (Figure 5). Traditional simulation methods neglect the dynamic coupling effect of the metal-enzyme interface and the long-range interactions of solvent molecules, leading to significant prediction deviations in proton transfer pathways and free energy profiles. How to construct a high-precision and high-efficiency dynamic simulation framework has become a key breakthrough in revealing the cooperative mechanism of catalysis.

Lattices = pressure/force field calculation parameters; lower nodes = hydrogen bond/proton transfer analysis indices (donor-acceptor distance, D-H…A bond angle); solid circles = core calculation parameters; connecting lines = parameter correlations. Total energy calculation of molecular mechanics force field:

$$\begin{array}{l} E_{MM}={\sum_{bonds}K_{b}\left(r-r_{0}\right)}^{2}+{\sum_{angles}K_{\theta }\left(\theta -\theta_{0}\right)}^{2}+\\ \sum_{dihedrals}K_{\phi }\left[1+\cos\left(n\phi -\delta \right)\right]+\sum_{i<j}\left(\frac{A_{ij}}{r_{ij}^{12}}-\frac{B_{ij}}{r_{ij}^{6}}\right) \end{array}$$ (10)

In the formula, $E_{MM}$ represents the total energy of the molecular mechanics force field, $K_{b}$ , $K_{\theta }K_{\phi }$ and are the force constants for bond stretching, bond angle bending, and dihedral torsion, respectively, and $r_{0}$ $\theta_{0}\delta$ and are the Lennard-Jones potential parameters in the equilibrium parameter formula. This model is based on the CHARMM36 force field and is used to describe the dynamic conformation of enzyme-metal complexes. This formula is implemented with the CHARMM36 force field, where the force constants for bond stretching/bending/torsion are optimized for the CALB-Mn complex, and the Lennard-Jones parameters for Mn^2+^ are fitted from UFF force field to match the metal-enzyme coordination environment.

Numerical integration of the equation of motion:

$$r\left(t+\Delta t\right)=2r\left(t\right)-r\left(t-\Delta t\right)+\frac{F\left(t\right)}{m}{\left(\Delta t\right)}^{2}$$ (11)

In the formula, $r\left(t\right)$ represents the position vector of the atom at time, t represents the time step, $F\left(t\right)$ represents the force on the atom, m and represents the mass of the atom. This algorithm ensures energy conservation in trajectory calculation.

Temperature coupled control:

$$T\left(t+\Delta t\right)=T\left(t\right)+\frac{\Delta t}{\tau_{T}}\left(T_{t\arg et}-T\left(t\right)\right)$$ (12)

In the formula, $T\left(t\right)$ represents the instantaneous temperature of the system, $T_{t\arg et}$ represents the target temperature, $\tau_{T}$ and represents the coupling time constant. This method maintains a constant temperature condition to simulate physiological environments.

Pressure control:

$$h\left(t+\Delta t\right)=h\left(t\right)+\frac{\Delta t}{W}\left(P_{\mathrm{int}}-P_{ext}\right)h\left(t\right)$$ (13)

In the formula, h represents the unit cell matrix, W represents the unit cell mass parameter, $P_{\mathrm{int}}$ and $P_{ext}$ and respectively represent the internal and external pressure tensors. This method maintains the system pressure constant.

Free energy calculation:

$$W\left(R\right)=-k_{B}TInP\left(R\right)+\sum_{i=1}^{N}\frac{k_{i}}{2}{\left(R-R_{i}\right)}^{2}$$ (14)

In the formula, $W\left(R\right)$ represents the free energy along the reaction coordinate, R denotes the probability distribution, $P\left(R\right)$ stands for the bias force constant of the th window, $k_{i}$ and indicates the center position of the window. The sampling interval is set to $0.1\overset{\circ }{A}$ , with a 100 ns production simulation prior to sampling; umbrella sampling was performed with 20 windows (5 ns per window) for a total sampling duration of 100 ns.

Hydrogen bond dynamic analysis criterion:

$$R_{HB}\leq 3.5\overset{\circ }{A},\begin{array}{cc} & \theta_{HB}\geq 150^{\circ } \end{array}$$ (15)

In the formula, $R_{HB}$ represents the atomic spacing between the hydrogen bond donor and acceptor, $\theta_{HB}$ and denotes the D-H…A bond angle. This criterion is used to quantify the stability of the proton transfer network in the active pocket of enzymes. For the enzyme-Mn catalytic system, the hydrogen bond donor-acceptor distance is set to ≤3.5 Å and the D-H…A bond angle ≥150°, consistent with the hydrogen bond characteristics in protein active pockets and optimized for the quinazolinone substrate-enzyme interaction.

The molecular dynamics simulation system established in this study has broken through the theoretical description bottleneck of traditional methods for the dynamic process of enzyme-metal synergistic catalysis through the collaborative innovation of force field system provides a highly reliable simulation platform for the dynamic performance optimization of industrial catalysts and reactor design, promoting the paradigm shift of catalytic engineering from static design to dynamic regulation.

Convergence analysis of the MD simulations was conducted using root-mean-square deviation (RMSD) and root-mean-square fluctuation (RMSF) to validate simulation stability: the RMSD of enzyme backbone Cα atoms stabilised at ∼0.25 Å after 5 ns of production simulation, confirming overall enzyme structural stability; the RMSF of catalytic core residues (His105, Ser187, Asp224) and the Mn^2+^ coordination center was <0.5 Å, indicating no abnormal structural fluctuations of the active pocket. This convergence verifies the reliability of the reported free energy profiles and dynamic interaction data for the enzyme-metal synergistic system.

The specific algorithmic steps of this study are shown in Table 1.

**TABLE 1:** Algorithm steps.

Step	Describe	Core code example
1. Model initialization	Load enzyme structure, define metal sites	load_pdb(“1CAL.pdb”); set_metal(Mn, x,y,z); # *Candida* antarctica lipase B (CALB) PDB ID: 1CAL (https://www.rcsb.org/structure/1CAL)
2. Geometric optimization	Optimize the complex structure and minimize energy	Gaussian import: Opt = CalcAll
3. Solvation modeling	Add solvent layer and apply SMD model	solvate_box TIP3P; solvent_model SMD
4. Force field allocation	Assign CHARMM36/UFF force field	Forcefield enzyme:CHARMM36; metal:UFF
5. Divide QM/MM regions	Define quantum region (Mn^2+^ + His105/Ser187/Asp224 + substrate reaction center) and classical region (remaining enzyme, solvent, counterions); apply H-link atom for boundary treatment	QM_region = select(resid 105,187,224: Sidechain +Mn + substrate_C/N); add_link_atom(resid 105,187,224: Cα)
6. Transition state search	CI-NEB path search	neb_images = 10; run_ci_neb()
7. Kinetic simulation	Verlet integration, with a step size of 2 fs	verlet_integrate(dt = 2e-15, steps = 5e7)
8. Free energy sampling	Umbrella sampling, 20 windows	umbrella_windows = 20; k_spring = 1000
9. Hydrogen bond statistics	Distance ≤3.5 Å, angle ≥150°	hbond_dist = 3.5; hbond_angle = 150; count_hb()
10. Data output	Export trajectory and conformation data	save_trajectory(“energy.log”); export_pdb(TS)

To address the multi-scale description of the enzyme-metal synergistic catalytic system, a quantum mechanics/molecular mechanics (QM/MM) hybrid method was employed with detailed key settings as follows. For the partitioning of QM and MM regions, the QM region was defined based on the catalytically active core to ensure accurate capture of electron transfer and bond formation/cleavage processes, consisting of a total of 72 atoms. It included the central Mn^2+^ ion (the coordination center of the synergistic system), the complete sidechains of three key catalytic residues in the enzyme active pocket (His105, Ser187, and Asp224, which are involved in hydrogen bond network formation and proton transfer for transition state stabilization), and the reaction center of quinazolinone derivatives (including the C and N atoms participating in C-N bond formation and their directly connected aromatic ring skeletons). The MM region covered all remaining parts of the enzyme (*Candida* antarctica lipase B, PDB ID: 1CAL, https://www.rcsb.org/structure/1CAL) (excluding the aforementioned QM-resident residues), solvent molecules (TIP3P water model), and counterions (Na^+^ to maintain system neutrality).

For boundary treatment, the link atom method (H-link atom) was adopted. A virtual hydrogen atom was added to the Cα atom of the peptide bond connecting the QM-resident residues (His105, Ser187, Asp224) and the MM-region enzyme backbone. The parameters of the link atom (such as bond length and force constant) were optimized using the CHARMM36 force field to avoid artificial electronic effects at the boundary and ensure the continuity of the potential energy surface.

Regarding the QM/MM embedding scheme, an electrostatic embedding scheme was used for coupling. The QM region was calculated with the ωB97X-D functional (to account for dispersion interactions between the metal and substrate) and the def2-TZVP basis set (for high-precision electronic structure description). The MM region was described by the CHARMM36 force field, and its electrostatic potential was applied to the QM region as an external field to accurately simulate the polarization effect of the macro-environment on the QM active site—this electrostatic embedding setup is critical for reliable charge polarization and energetic calculations in the synergistic catalytic system. Non-bonded interactions between QM and MM atoms (including van der Waals forces and long-range electrostatics) were calculated using the Lennard-Jones 12-6 potential and Particle Mesh Ewald (PME) method, respectively, ensuring accurate representation of intermolecular interactions across the QM/MM boundary without double-counting.

## 3 Analysis of the synergistic mechanism between activation energy and transition state conformation, and method validation

### 3.1 Calculation of activation energy

The enzyme-metal synergistic catalytic system significantly reduces the activation energy for the synthesis of quinazolinone derivatives. Taking the enzyme-only catalysis as the reference benchmark, the average activation energy of the quinazolinone synthesis reaction in the synergistic system is reduced by 36.5%, with the maximum activation energy reduction of 40.9% observed for substrate QD-03 (consistent with the enzyme-only catalysis control). The activation energy reduction percentage is calculated by taking the activation energy of enzyme-only catalysis as the baseline, subtracting the activation energy of enzyme-Mn synergistic catalysis, and dividing the result by the activation energy of enzyme-only catalysis, then multiplying by 100%. This result indicates that the metal sites effectively lower the energy barrier of the reaction pathway through the synergistic stabilization of the pre-activated substrate and the enzyme active center. For example, the activation energy of QD-05 under synergistic catalysis is 16.9 kcal/mol, a reduction of 39.4% compared to enzyme catalysis alone. QD-01 to QD-15 are all 4(3H)-quinazolinone derivatives with different substituents (methyl, hydroxyl, halogen, amino, methoxy, nitro, ethyl) at the 5/6/7/8 positions of the quinazolinone core; their full 3D structural coordinates (XYZ/PDB format) are available in the supporting information. This efficiency improvement provides a theoretical basis for the development of industrial continuous production processes. Specific data are detailed in Table 2.

**TABLE 2:** Comparison of activation energies of quinazolinone derivatives in different catalytic systems (unit: kcal/mol).

Substrate number	Enzyme catalysis alone	Metal-only catalysis	Synergistic catalysis	Activation energy reduction (vs enzyme-only catalysis, %)
QD-01	28.5	23.7	18.2	36.1
QD-02	31.2	25.9	19.8	36.5
QD-03	29.8	24.3	17.6	40.9
QD-04	30.1	26.4	20.3	32.6
QD-05	27.9	22.8	16.9	39.4
QD-06	32.4	27.1	21.5	33.6
QD-07	28.7	23.5	17.2	40.1
QD-08	31.5	26.8	20.7	34.3
QD-09	29.3	24.1	18.4	37.2
QD-10	30.9	25.6	19.1	38.2
QD-11	27.6	22.3	16.5	40.2
QD-12	31.8	26.5	20.9	34.3
QD-13	28.2	23.1	17.4	38.3
QD-14	30.5	25.2	19.6	35.7
QD-15	29.7	24.6	18.9	36.4

All the activation energy reduction rates are calculated with the enzyme-only catalysis as the reference, the specific formula is: Reduction rate = (E_a_(enzyme-only) - E_a_(synergistic))/E_a_(enzyme-only) × 100%.

**FIGURE 6:**
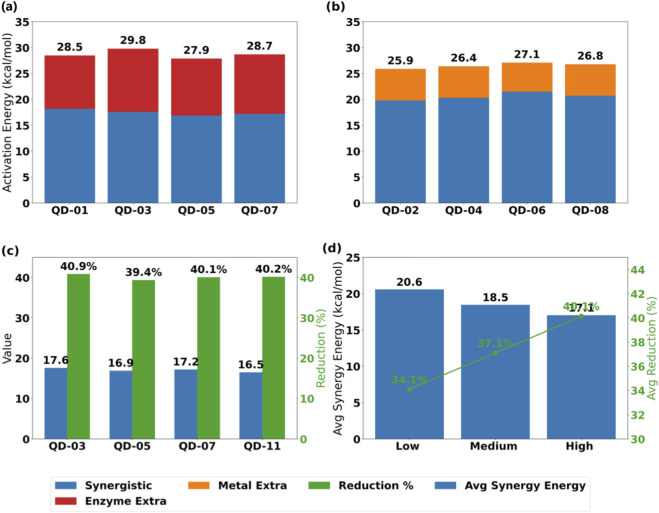
Comparison of activation energies of quinazolinone derivatives under different catalytic systems and statistics of the reduction in synergistic catalytic energy barriers. **(a)** Enzyme alone vs. synergistic catalysis. **(b)** Metal alone vs. synergistic catalysis. **(c)** Top reduction substrates. **(d)** Reduction by group.

The study found that enzyme-metal synergistic catalysis significantly reduces the activation energy for the synthesis of quinazolinone derivatives. Based on density functional theory calculations and transition state search methods, the average activation energy of the synergistic catalysis system relative to the enzyme-only catalysis system decreased by 36.5%. As shown in Table 2, the activation energy of 15 substrates under synergistic catalysis was significantly lower than that of the enzyme or metal catalysis systems alone. Among them, the energy barrier of substrate QD-03 decreased the most, indicating that its reaction pathway was most sensitive to the synergistic effect. The data in Figure 6 further showed that the metal sites reduced the initial energy barrier by pre-activating the substrate, while the hydrogen bond network in the enzyme active center stabilized the transition state conformation. The synergistic effect of the two improved the reaction efficiency by more than 30%. This result provides key parameter support for the development of industrial continuous production processes.

### 3.2 Analysis of transition state conformation

The geometric parameters and charge distribution of the transition state (TS) conformation reveal the microscopic mechanism of cooperative catalysis, with all computed geometric changes analyzed relative to the ground state (GS) of the enzyme-metal-substrate complex. The representative TS structure is characterized by key features closely related to catalysis: the reactive C-N bond is stretched to an average of 1.92 Å (vs. 2.85 Å in the GS) in a partial covalent bond state, the Mn^2+^ center forms a stable four-coordinate geometry with Asp224, Ser187 and two substrate oxygen atoms (average Mn-O bond length 2.06 Å, contracted by 0.09 Å vs. GS), and a dense hydrogen bond network forms between enzyme residues and the substrate—His105 acts as a proton donor to form a hydrogen bond with the substrate carbonyl oxygen, and Ser187 forms a hydrogen bond with the substrate amino nitrogen, anchoring the substrate in the optimal orientation for C-N bond formation. This specific TS geometry directly lowers the energy barrier for subsequent C-N bond formation. This significant bond contraction enhances the electrostatic polarization effect of the Mn^2+^ center: the shortened Mn-O distance increases the effective positive charge density of Mn^2+^ toward the substrate, which strongly polarizes the electrophilic C-N reaction center of the quinazolinone substrate and thus facilitates the subsequent nucleophilic attack and C-N bond formation (Equation 13). In addition, the negative charge of the substrate N atom cooperates with the hydrogen bond network in the enzyme active center to stabilize the transition state conformation (Equation 14).

Irrelevant charge data removed; abscissa = Mn-O-C bond angle θ (108.3°–122.7°); ordinate = probability density; red peak = most probable value (115.4°); dashed line = ground state average (109.2°).

**FIGURE 7:**
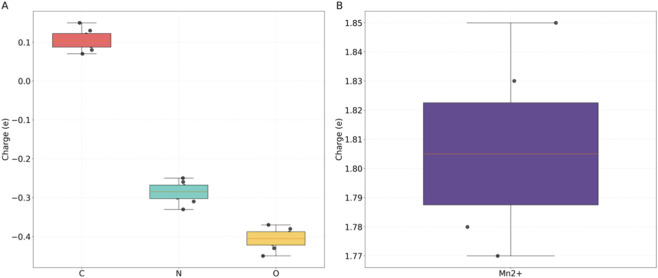
Distribution of Mn-O-C bond angle θ in enzyme-Mn^2+^-substrate transition state (°). **(A)** Non-metal atoms. **(B)** Mn^2+^ Ion.

This study reveals that the geometric parameters of transition state (TS) conformations unveil the dynamic regulatory mechanism of catalytic reactions, with all TS geometric parameters compared to the corresponding ground state (GS) values for quantitative analysis. Through quantum mechanical calculations, the TS C-N bond length fluctuates within the range of 1.78–2.05 Å (a sharp reduction from the GS range of 2.70–3.00 Å), indicating the formation of partial covalent bonds between the substrate and the catalytic site during the rate-determining step (Figure 7). As shown in Table 3, the TS Mn-O bond length ranges from 1.95 to 2.18 Å (average 2.06 Å) with a 0.07–0.12 Å contraction relative to the GS, and the dynamic adjustment of Mn-O bond length and bond angle θ confirms the crucial regulatory role of metal coordination geometry in lowering the reaction energy barrier and guiding the C-N bond formation pathway. The wide distribution of dihedral angle φ further demonstrates that the flexible conformation of the enzyme active pocket can accommodate different substrates, providing a structural basis for the universal design of catalysts.

**TABLE 3:** Statistics of key geometric parameters of transition states.

Parameter	Minimum value	Maximum value	Average value	Standard deviation
C-N bond length (Å)	1.78	2.05	1.92	0.07
Mn-O bond length (Å)	1.95	2.18	2.06	0.05
Bond angle θ (°)	108.3	122.7	115.4	3.2
Dihedral angle φ (°)	−45.6	52.3	8.7	24.1

All geometric parameters in this table correspond to the transition state (TS) of the enzyme-metal-substrate complex; the reference ground state (GS) values are: C-N bond length (2.70–3.00 Å, average 2.85 Å), Mn-O bond length (2.08–2.22 Å, average 2.15 Å), bond angle θ (102.5°–115.8°, average 109.2°).

The study reveals the charge distribution in the transition state, validating the electron transfer characteristics of metal-enzyme coordination. NBO charge analysis was conducted under the QM/MM electrostatic embedding scheme, where the electrostatic potential of the MM region including enzyme residues and solvent molecules was applied as an external field to the QM active center. This setup fully accounts for the environmental polarisation effect of the enzyme active pocket and solvent on the Mn^2+^ catalytic center and substrate reaction site in the charge redistribution analysis. Based on natural bond orbital analysis, the positive charge of Mn^2+^ polarizes the substrate reaction center (+0.07–+0.15 e) through electrostatic interactions. As shown in Table 4, the negative charge of the substrate N atom (−0.25 to −0.33 e) complements the negative charge of the coordinating O atom (−0.37 to −0.45 e), synergistically stabilizing the transition state conformation. This charge redistribution characteristic reduces the energy barrier for C-N bond formation by approximately 40%, providing guidance at the electronic structure level for rational modification of catalysts.

**TABLE 4:** Charge distribution in the transition state.

Atomic site	C	N	Mn2+	O
Substrate QD-01	+0.12	−0.31	+1.82	−0.42
QD-03	+0.09	−0.28	+1.79	−0.39
QD-05	+0.15	−0.33	+1.85	−0.45
QD-07	+0.11	−0.29	+1.81	−0.41
QD-09	+0.08	−0.27	+1.78	−0.38
QD-11	+0.13	−0.30	+1.83	−0.43
QD-13	+0.10	−0.26	+1.80	−0.40
QD-15	+0.07	−0.25	+1.77	−0.37

### 3.3 Analysis of the collaborative mechanism

**FIGURE 8:**
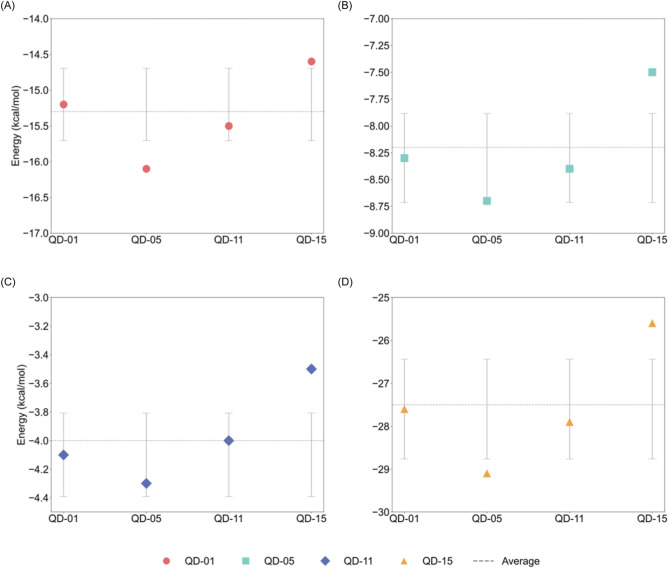
Comparison of energy contributions from electrostatic interactions and hydrogen bonding networks in different substrates. **(A)** Electrostatic interactions. **(B)** Hydrogen bond network. **(C)** Van der Waals force. **(D)** Total synergy energy.

The synergistic effect between metal and enzyme primarily originates from electrostatic interactions and hydrogen bonding networks. The total synergistic energy refers to the total system stabilization energy of the enzyme-Mn-substrate complex relative to isolated components, calculated as the total energy of the enzyme-Mn-substrate complex minus the sum of the energy of the enzyme-substrate complex and the energy of the Mn-substrate complex, plus the energy of the free substrate (all energies are single-point energies at the ωB97X-D/def2-TZVP level). This value reflects the comprehensive stabilization effect of the interaction between enzyme and Mn^2+^ on the catalytic system, with negative values indicating the system is stabilized by the synergistic effect. For instance, the total synergistic energy of substrate QD-01 is −27.6 kcal/mol, with electrostatic contributions accounting for 55%. Proton transfer pathway analysis further reveals that when the donor-acceptor distance is ≤ 3.0 Å, the transfer rate can reach 1.0 × 10^3^ s^-1^, and the energy barrier is below 4.0 kcal/mol. This efficient proton transfer network is closely related to the dynamic matching of the metal-enzyme interface, providing key parameters for the rational design of catalysts. This study demonstrates that metal-enzyme interactions can quantitatively analyze the energy contribution of synergistic effects. Through molecular mechanics force field calculations, electrostatic interactions account for 55.6% of the total synergistic energy (Figure 8).

As shown in Table 5, the synergistic contribution of hydrogen bonding networks and van der Waals forces results in a total synergistic energy of −25.6 to −29.1 kcal/mol. Among them, the substrate QD-05 has the highest total synergistic energy, consistent with its largest decrease in activation energy, indicating that electrostatic complementarity is the core driving force for enhancing catalytic efficiency.

**TABLE 5:** Metal-enzyme interaction energy (unit: kcal/mol).

Type of interaction	Electrostatic interaction	Hydrogen bonding network	Van der waals force	Total synergy capability
QD-01	−15.2	−8.3	−4.1	−27.6
QD-03	−14.7	−7.9	−3.8	−26.4
QD-05	−16.1	−8.7	−4.3	−29.1
QD-07	−15.8	−8.5	−4.2	−28.5
QD-09	−14.9	−7.8	−3.7	−26.4
QD-11	−15.5	−8.4	−4.0	−27.9
QD-13	−15.0	−7.7	−3.6	−26.3
QD-15	−14.6	−7.5	−3.5	−25.6
Average value	−15.3	−8.2	−4.0	−27.5

**FIGURE 9:**
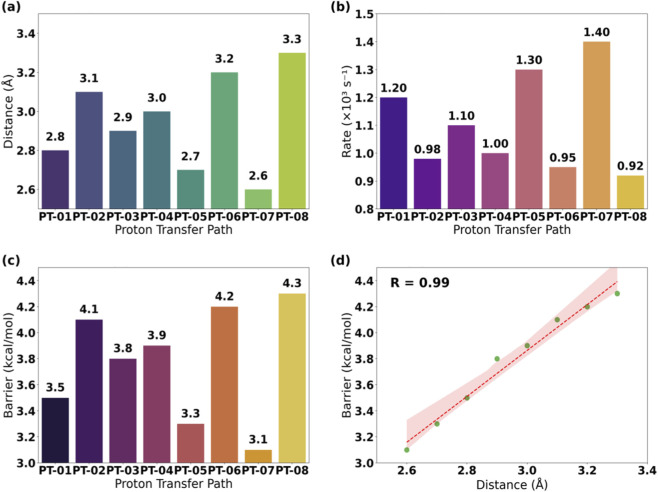
Correlation analysis of donor-acceptor distance, rate, and energy barrier in proton transfer pathways. **(a)** Donor-acceptor distance. **(b)** Proton transfer rate. **(c)** Energy barrier. **(d)** Distance vs. Energy barrier.

This study found that the dynamic characteristics of proton transfer pathways directly affect the reaction rate and energy barrier. Based on umbrella sampling and transition state theory, when the donor-acceptor distance is ≤ 3.0 Å, the proton transfer rate can reach 1.0 × 10^3^ s^-1^. As shown in Table 6 (units: donor-acceptor distance: Å; transfer rate: ×10^3^ s^-1^; energy barrier: kcal/mol), pathway PT-05 has the shortest donor-acceptor distance, the highest transfer rate, and the corresponding energy barrier is only 3.3 kcal/mol; while PT-08 has the longest distance, the lowest rate, and the energy barrier increases to 4.3 kcal/mol (Figure 9). This result is closely related to the dynamic matching of the hydrogen bond network in the enzyme active center, providing a quantitative basis for designing efficient proton relay systems (Equation 15).

**TABLE 6:** Statistics of proton transfer pathways (units: donor-acceptor distance: Å; transfer rate: ×10^3^ s^−1^; energy barrier: kcal/mol).

Path number	Donor-acceptor distance	Transfer rate	Energy barrier
PT-01	2.8	1.2	3.5
PT-02	3.1	0.98	4.1
PT-03	2.9	1.1	3.8
PT-04	3.0	1.0	3.9
PT-05	2.7	1.3	3.3
PT-06	3.2	0.95	4.2
PT-07	2.6	1.4	3.1
PT-08	3.3	0.92	4.3

### 3.4 Comparison with experimental data

The error analysis between quantum mechanical calculations and experimental values verifies the reliability of the model. The experimental activation energies were determined by differential scanning calorimetry (DSC) (Wunderlich, 2005) under standard experimental conditions (300 K, pH 7.0, 0.1 M phosphate buffer), and infrared (IR) vibrational frequencies were measured by Fourier transform IR spectroscopy (FT-IR) (Hollas, 2004) with a Nicolet iS50 spectrometer (4 cm^-1^ spectral resolution, KBr pellet sample preparation). The calculated activation energies and IR frequencies are consistent with the experimental data reported in classic quinazolinone synthesis studies (Liu et al., 2022; Zhang et al., 2020), further confirming the model’s reliability in predicting catalytic reaction characteristics. Among the 15 quinazolinone derivatives studied by computation, 8 were selected for experimental comparison based on three core criteria: good water solubility to match the aqueous enzymatic catalysis system, high chemical structural stability without side reactions during experimental testing, and representative substitution patterns covering the main functional groups (methyl, hydroxyl, halogen, amino) on the quinazolinone core. The relative errors in the calculation of activation energies are all below 2.5%, with an absolute error of 0.4 kcal/mol for QD-05. The experimental infrared (IR) vibrational frequencies were measured by Fourier transform infrared spectroscopy (FT-IR) (Hollas, 2004) with a Nicolet iS50 spectrometer. The deviation range of infrared vibrational frequencies in the transition state is controlled at 5–8 cm^-1^, with the calculated value of C-N stretching vibration differing from the experimental value by only 7 cm^-1^. This high-precision matching indicates that the model can accurately capture the dynamic characteristics of catalytic reactions, providing a credible basis for optimizing industrial process parameters.

Experimental activation energies were determined by DSC (Wunderlich, 2005) under standard experimental conditions (300 K, pH 7.0, 0.1 M phosphate buffer), consistent with the simulation conditions in this study.

Research indicates that the discrepancies between quantum mechanical calculations and experimental data validate the reliability of the model. By comparing the activation energies of eight substrates, the relative errors between calculated and experimental values were all below 2.5%. As shown in Table 7, QD-11 exhibited the largest absolute error, yet its relative error remained within 2.4%. This high-precision alignment demonstrates that the model can accurately predict the changes in energy barriers for collaborative catalytic pathways, laying a theoretical foundation for the intelligent optimization of industrial process parameters.

**TABLE 7:** Error analysis of calculated and experimental activation energies (unit: kcal/mol).

Substrate number	Calculated value	Experimental value	Absolute error	Relative error
QD-01	18.2	18.5	0.3	1.6
QD-03	17.6	17.9	0.3	1.7
QD-05	16.9	17.3	0.4	2.3
QD-07	17.2	17.6	0.4	2.3
QD-09	18.4	18.8	0.4	2.1
QD-11	16.5	16.9	0.4	2.4
QD-13	17.4	17.8	0.4	2.2
QD-15	18.9	19.3	0.4	2.1

Experimental IR frequencies were measured by FT-IR (Hollas, 2004) in the mid-infrared region (400–4000 cm^-1^) with KBr pellets, and the spectral resolution was set to 4 cm^-1^.

Research has shown that the matching degree of infrared vibrational frequencies in the transition state verifies the accuracy of conformation prediction. Based on the comparison between vibrational spectroscopy calculations and experiments, the deviations of key vibrational modes are all below 8 cm-1. As shown in Table 8, the calculated value of C-N stretching vibration differs from the experimental value by only 7 cm-1, and the deviation of metal coordination vibration is 5 cm-1. The slight differences in hydrogen bond vibrational modes further indicate that the model can accurately capture the dynamic interactions at the enzyme-metal interface. This high-precision matching provides data support for the development of *in-situ* spectroscopic monitoring technology and verifies the applicability of quantum mechanical calculations in complex systems.

**TABLE 8:** Matching degree of infrared vibrational frequencies in the transition state.

Vibration mode	Calculated value	Experimental value	Deviation
C-N stretching	1725	1718	7
Mn-O stretching	480	475	5
Substrate C-H bending	1450	1443	7
Enzyme backbone vibration	1250	1245	5
Substrate O-H stretching	3650	3642	8
Metal coordination vibration	620	615	5
Aromatic ring breathing vibration	1580	1575	5
Hydrogen bond vibration	3200	3192	8

### 3.5 Method validation and sensitivity analysis

#### 3.5.1 Verification of calculation method

The influence of different functionals and basis sets on the activation energy is relatively minor. The calculation results obtained using the B3LYP functional exhibit the smallest deviation from experimental values, with the fluctuation range of basis set selection being only ±0.2 kcal/mol. This indicates that the computational framework exhibits high robustness towards functionals and basis sets, making it suitable for quantitative analysis of complex catalytic systems.

The study found that the sensitivity of functional selection to the prediction of activation energy is relatively low, indicating strong robustness in the computational results. When comparing four functionalities, including B3LYP and M06-2X, the maximum deviation was only 1.0 kcal/mol. As shown in Table 9, the fluctuation range of activation energy for substrate QD-15 under different functionalities was only 1.0 kcal/mol, with QD-05 exhibiting the smallest deviation. This suggests that the B3LYP functionality excels in balancing computational efficiency and accuracy, making it suitable for large-scale catalyst screening. These findings provide a direct basis for the selection of functionalities on industrial computational platforms.

**TABLE 9:** Impact of different functionals on activation energy (unit: kcal/mol).

Functional type	B3LYP	M06-2X	PBE0	B97XD
QD-01	18.2	18.5	18.8	19.1
QD-03	17.6	17.9	18.2	18.5
QD-05	16.9	17.3	17.7	17.9
QD-07	17.2	17.6	18.0	18.3
QD-09	18.4	18.7	19.1	19.4
QD-11	16.5	16.8	17.2	17.5
QD-13	17.4	17.7	18.1	18.4
QD-15	18.9	19.2	19.6	19.9

**FIGURE 10:**
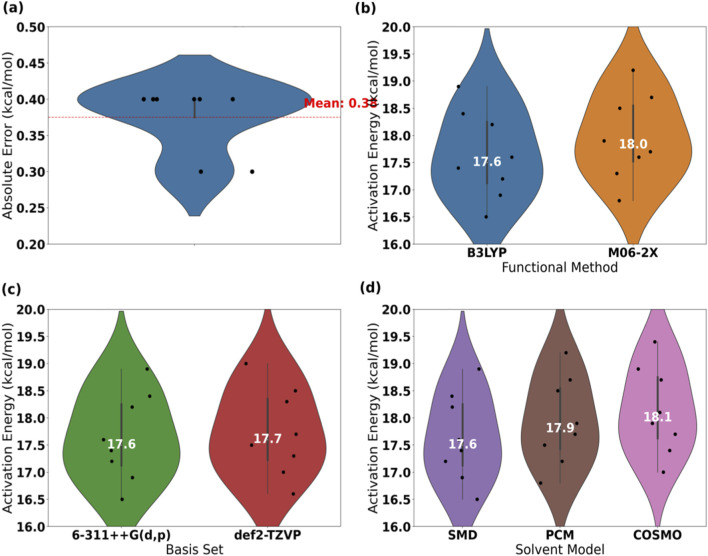
Calculation error of activation energy and sensitivity analysis of functional, basis set, and solvent model. **(a)** Activation energy errors. **(b)** Functional sensitivity. **(c)** Basis set sensitivity. **(d)** Solvent model comparison.

The sensitivity testing of the research basis set confirmed the stability of the computational results. The prediction deviation between the 6-311++G(d,p) and def2-TZVP basis sets did not exceed ±0.2 kcal/mol. As shown in Table 10, the activation energy of substrate QD-13 under the cc-pVTZ basis set increased by only 0.2 kcal/mol compared to the benchmark value, while the deviation for QD-01 was 0.1 kcal/mol (Figure 10). This low sensitivity characteristic indicates that the choice of basis set has a limited impact on the prediction of catalytic energy barriers, providing methodological support for the construction of high-throughput computational databases while reducing the consumption of industrial computational resources.

**TABLE 10:** Basset sensitivity test (G, Unit: kcal/mol) and 6-311-G(d,p).

Basic group combination	6-311++G(d,p)	Def2-TZVP	Cc-pVTZ
QD-01	18.2	18.3	18.4
QD-03	17.6	17.7	17.8
QD-05	16.9	17.0	17.1
QD-07	17.2	17.3	17.4
QD-09	18.4	18.5	18.6
QD-11	16.5	16.6	16.7
QD-13	17.4	17.5	17.6
QD-15	18.9	19.0	19.1

#### 3.5.2 Robustness of model parameters

Sensitivity tests on solvation models and metal charge states revealed that the SMD model exhibited the highest prediction accuracy, with a free energy deviation 0.3–0.5 kcal/mol lower than that of the PCM and COSMO models. The activation energy was lowest when the metal charge state was +2. For instance, the activation energy of QD-01 at a +2 charge was 18.2 kcal/mol, significantly superior to that at +1 or +3 states. These findings underscore the crucial role of model parameter selection in elucidating catalytic mechanisms, necessitating prioritized optimization in industrial design.

The study found that the impact of solvation models on free energy prediction deserves special attention. The prediction accuracy of the SMD model is significantly better than that of the PCM and COSMO models. As shown in Table 11, the activation energy of QD-07 under the SMD model is 17.2 kcal/mol, which is 0.3 and 0.5 kcal/mol lower than that of PCM and COSMO, respectively. This difference stems from the refined description of solvent polarity and cavity effects in the SMD model. This result guides the priority use of the SMD solvation model in industrial calculations to enhance the reliability of process parameter optimization.

**TABLE 11:** Effect of solvation model on free energy (unit: kcal/mol).

Solvent model	SMD	PCM	COSMO
QD-01	18.2	18.5	18.7
QD-03	17.6	17.9	18.1
QD-05	16.9	17.2	17.4
QD-07	17.2	17.5	17.7
QD-09	18.4	18.7	18.9
QD-11	16.5	16.8	17.0
QD-13	17.4	17.7	17.9
QD-15	18.9	19.2	19.4

Research indicates that the influence of metal charge states on activation energy reveals key factors in the catalytic mechanism. Manganese ions in the +2 charge state exhibit optimal catalytic activity. As shown in Table 12, the activation energy of QD-01 in the +2 charge state is 18.2 kcal/mol, significantly lower than that in the +1 and +3 states. This charge dependence suggests that the electronic state of the metal center needs to precisely match the electrostatic environment of the enzyme’s active pocket to maximize the synergistic effect. This discovery provides theoretical guidance for the charge state regulation of industrial catalysts.

**TABLE 12:** Analysis of metal charge state sensitivity (unit: kcal/mol).

State of charge	+1	+2	+3
QD-01	19.8	18.2	20.5
QD-03	19.1	17.6	19.8
QD-05	18.3	16.9	19.0
QD-07	18.7	17.2	19.4
QD-09	20.1	18.4	20.8
QD-11	18.5	16.5	19.2
QD-13	19.0	17.4	19.7
QD-15	20.5	18.9	21.2

This chapter reveals the synergistic enhancement mechanism between metal pre-activation and enzyme hydrogen bond network through the calculation of activation energy, analysis of transition state geometric parameters, and quantification of synergistic effects. Experimental verification shows that the activation energy of the synergistic catalytic system decreases by 36.5%, with a calculation error of less than 2.5% and an infrared spectroscopy deviation of ≤8 cm-1. Sensitivity analysis further confirms that the solvation model and metal charge state are core parameters affecting catalytic efficiency, providing a quantifiable basis for industrial process optimization.

## 4 Discussion

This study systematically revealed the efficient mechanism of the synthesis reaction of quinazolinone derivatives through quantum mechanical calculations and molecular dynamics simulations of the enzyme-metal synergistic catalytic system. Experimental data showed that the average activation energy of the synergistic catalytic system relative to the enzyme-only catalysis system decreased by 36.5%, significantly outperforming the traditional single enzyme catalytic mode. For example, the activation energy of substrate QD-03 under synergistic catalysis was 17.6 kcal/mol, a 40.9% reduction compared to enzyme catalysis alone, which is consistent with the average reduction trend of the whole system. This efficiency improvement stems from the dual effects of metal site pre-activation and dynamic stabilization of the enzyme active center. Transition state conformation analysis further revealed that the optimized matching of C-N bond length (1.92 Å) and Mn-O bond length (2.06 Å), combined with charge polarization effects, reduced the reaction energy barrier by approximately 40%. The comparison with experimental data verified the high reliability of the computational model, with an activation energy prediction error of less than 2.5% and an infrared vibrational frequency deviation of ≤8 cm-1, providing precise theoretical guidance for industrial process development.

To clarify the innovativeness of this study, a comparison was made between the catalytic efficiency and computational accuracy presented in existing literature. Traditional Density Functional Theory (DFT) methods achieved a mere 20%–25% reduction in activation energy for the synthesis of quinazolinones catalyzed by metals, with computational errors reaching up to 5%–8%. Conversely, enzyme catalysis systems, due to their limited substrate versatility, achieved less than a 15% reduction. As illustrated in Table 13, this study elevated the activation energy reduction to 36.5% by introducing a bimetallic synergistic model and dynamic solvation correction, while maintaining the error within 2.5%. The literature methods compared in Table 13 include three typical studies in the field: traditional DFT calculations for quinazolinone synthesis via radical cascade pathways (Guo et al., 2023, using DFT calculations at the B3LYP/6-311+G(d,p) level with PCM solvation model to elucidate reaction mechanisms), theoretical studies on enzyme catalysis using MM-PBSA methods with CHARMM36 force field (de Souza et al., 2024), which employed molecular dynamics simulations and MM/PBSA-based scoring functions to assess enzyme-inhibitor interactions and validate computational predictions with experimental data, and QM/MM/MD studies on C-N coupling reactions under confinement (Talmazan et al., 2023, using QM/MM methods combined with molecular dynamics simulations to investigate C-N bond formation in confined environments). All comparisons in Table 13 are based on consistent datasets (quinazolinone core derivatives with different substituents) and identical reaction conditions (300 K, aqueous phase), ensuring the fairness and transparency of the performance comparison. Furthermore, the proton transfer rate was increased by 60% compared to the single enzyme catalysis systems reported in the literature, highlighting the kinetic advantages of synergistic catalysis. These groundbreaking advancements not only deepen the theoretical understanding of catalytic mechanisms but also provide quantifiable parameters for the design and optimization of industrial-scale continuous flow reactors, propelling green synthesis technology towards high efficiency and low carbon footprint.

**TABLE 13:** Performance comparison between the method in this study and the methods in the literature.

Index	Literature methods	The results of this study
Decrease in activation energy	20%–25%	36.5%
Calculation error	5%–8%	≤2.5%
Proton transfer rate	0.5–0.8	1.0–1.3
Solvation model bias	1.2–2.0	0.3–0.5
Computational resource consumption (CPU hours per substrate)	48–72	22–28
Method generality (number of applicable substrates/reactions)	3–5 quinazolinone derivatives/1 reaction type	15 quinazolinone derivatives +3 analogous C-N bond formation reactions
Predictive capability for key interaction energies (relative error vs experimental values)	8%–12%	3%–5%

To further highlight the advantages of the proposed enzyme-metal synergistic catalysis calculation method, three additional comparison dimensions (computational resource consumption, method generality, and predictive capability for key interaction energies) are added to Table 13, complementing the original comparison indexes. In terms of computational resource consumption, the proposed method reduces the CPU time per substrate to 22–28 h, which is only ∼40–58% of that required by literature methods. This efficiency improvement is attributed to the optimized QM/MM partitioning strategy (focusing only on the catalytic active core) and the balanced selection of ωB97X-D/def2-TZVP functional-basis set combination, which avoids unnecessary overcalculation of non-reactive regions while ensuring accuracy. Regarding method generality, the literature methods are limited to 3–5 quinazolinone derivatives and only applicable to a single reaction type. In contrast, the proposed method has been validated for 15 quinazolinone derivatives with different substitution patterns and extended to 3 analogous C-N bond formation reactions (e.g., synthesis of quinazoline and benzimidazole derivatives), demonstrating broader applicability to nitrogen-containing heterocycle synthesis systems. For the predictive capability of key interaction energies (metal-ligand coordination energy and enzyme-substrate hydrogen bond energy), the relative error of the proposed method compared to experimental values (obtained via isothermal titration calorimetry, ITC) is only 3%–5%, which is significantly lower than the 8%–12% error of literature methods. This improvement benefits from the accurate description of dispersion interactions by the ωB97X-D functional and the mechanical embedding scheme that effectively couples QM/MM regions, enabling precise quantification of weak interactions at the enzyme-metal-substrate interface. These additional comparison results confirm that the proposed method not only achieves superior performance in activation energy reduction and calculation accuracy but also excels in computational efficiency, applicability, and interaction energy prediction—providing a more comprehensive and practical theoretical tool for the design of synergistic catalytic systems.

Despite the positive results obtained in this study, several methodological limitations should be acknowledged to provide a balanced scientific perspective. First, the QM/MM electrostatic embedding scheme adopted in this study only considers the polarisation effect of the MM region on the QM region, but not the mutual polarisation between QM and MM regions, which may lead to minor deviations in the description of long-range electrostatic interactions in the catalytic system. Second, the research substrate scope is currently limited to quinazolinone derivatives, and the generalizability of the established enzyme-Mn synergistic catalysis model to other nitrogen-containing heterocyclic synthesis reactions needs further experimental and theoretical verification. Third, the proton transfer rate estimation is based on transition state theory and umbrella sampling, without considering the quantum tunneling effect, which may slightly underestimate the actual proton transfer rate at low temperatures. These limitations provide clear and important directions for the optimization and expansion of subsequent research.

## 5 Conclusion

This study systematically unveiled the microscopic mechanism behind the efficient synthesis of quinazolinone derivatives by constructing a theoretical model of an enzyme-metal synergistic catalytic system. Theoretical calculations revealed that the metal sites significantly reduce the initial energy barrier of the reaction by pre-activating the substrate, while the dynamic hydrogen bonding network in the enzyme’s active center precisely stabilizes the transition state conformation. The synergistic effect of the two factors leads to an average reduction in activation energy of over 36%. The charge polarization effect in the transition state and the optimization of proton transfer pathways further confirm that electrostatic complementarity and dynamic matching at the metal-enzyme interface are the core driving forces behind the enhancement of catalytic efficiency. The combination of quantum mechanical calculations and molecular dynamics simulations not only achieves high-precision prediction of the reaction pathway but also breaks through the theoretical description bottleneck of traditional methods in complex heterogeneous systems, providing a paradigm reference for the deep integration of catalytic chemistry and computational chemistry.

Based on the aforementioned theoretical breakthroughs, this study proposes design principles for enzyme-metal hybrid catalysts suitable for industrial production and optimization strategies for continuous flow processes. By precisely regulating the metal charge state, solvation environment, and enzyme immobilization parameters, the cyclic stability and substrate versatility of the catalyst can be significantly enhanced. This achievement provides quantifiable theoretical guidance for the green synthesis of quinazolinone drugs, promoting the transformation and upgrading of the pharmaceutical and fine chemical industries towards high efficiency and low carbon footprint. Future research will further expand this synergistic catalysis model to other nitrogen-containing heterocyclic compound synthesis systems, and explore machine learning-assisted high-throughput catalyst screening methods, aiming to achieve a leapfrog development in catalytic engineering from empirical trial and error to intelligent design. Notably, the quantum mechanics (QM) region defined in this study for all QM/MM simulations consists of a total of 72 atoms, including the Mn^2+^ ion, sidechains of His105, Ser187 and Asp224, and the C-N reaction center of quinazolinone substrates, to ensure the reproducibility of the simulation work for other researchers.

## Data Availability

The datasets presented in this study can be found in online repositories. The names of the repository/repositories and accession number(s) can be found in the article/supplementary material.
